# Supplementary material for: Bioactive Compounds from the Mushroom-Forming Fungus Chlorophyllum molybdites
Source: Antibiotics (Basel). 2023 Mar 16;12(3):596. doi: 10.3390/antibiotics12030596 (PMC10044768; doi:10.3390/antibiotics12030596)
Supplement: Supplementary file 1 [file antibiotics-12-00596-s001.zip › antibiotics-2248094-supplementary.pdf]

Supplementary Materials

Bioactive Compounds from the Mushroom-Forming Fungus *Chlorophyllum molybdites*

Jing Wu [1,2], Takeru Ohura [3], Ryuhei Ogura [3], Junhong Wang [4], Jae-Hoon Choi [1,2,3,4,5], Hajime Kobori [2,6], Corina N. D'Alessandro-Gabazza [7], Masaaki Toda [7], Taro Yasuma [7], Esteban C. Gabazza [7], Yuichi Takikawa [1], Hirofumi Hirai [1,2,3,4,5] and Hirokazu Kawagishi [1,2,*]

[1] Faculty of Agriculture, Shizuoka University, 836 Ohya, Suruga-ku, Shizuoka 422-8529, Japan
[2] Research Institute for Mushroom Science, Shizuoka University, 836 Ohya, Suruga-ku, Shizuoka 422-8529, Japan
[3] Graduate School of Integrated Science and Technology, Shizuoka University, 836 Ohya, Suruga-ku, Shizuoka 422-8529, Japan
[4] Graduate School of Science and Technology, Shizuoka University, 836 Ohya, Suruga-ku, Shizuoka 422-8529, Japan
[5] Research Institute of Green Science and Technology, Shizuoka University, 836 Ohya, Suruga-ku, Shizuoka 422-8529, Japan
[6] Iwade Research Institute of Mycology Co., Ltd., Suehirocho 1-9, Tsu 514-0012, Japan
[7] Department of Immunology, Mie University Graduate School of Medicine, Edobashi 2-174, Tsu 524-8507, Japan

* Correspondence: kawagishi.hirokazu@shizuoka.ac.jp

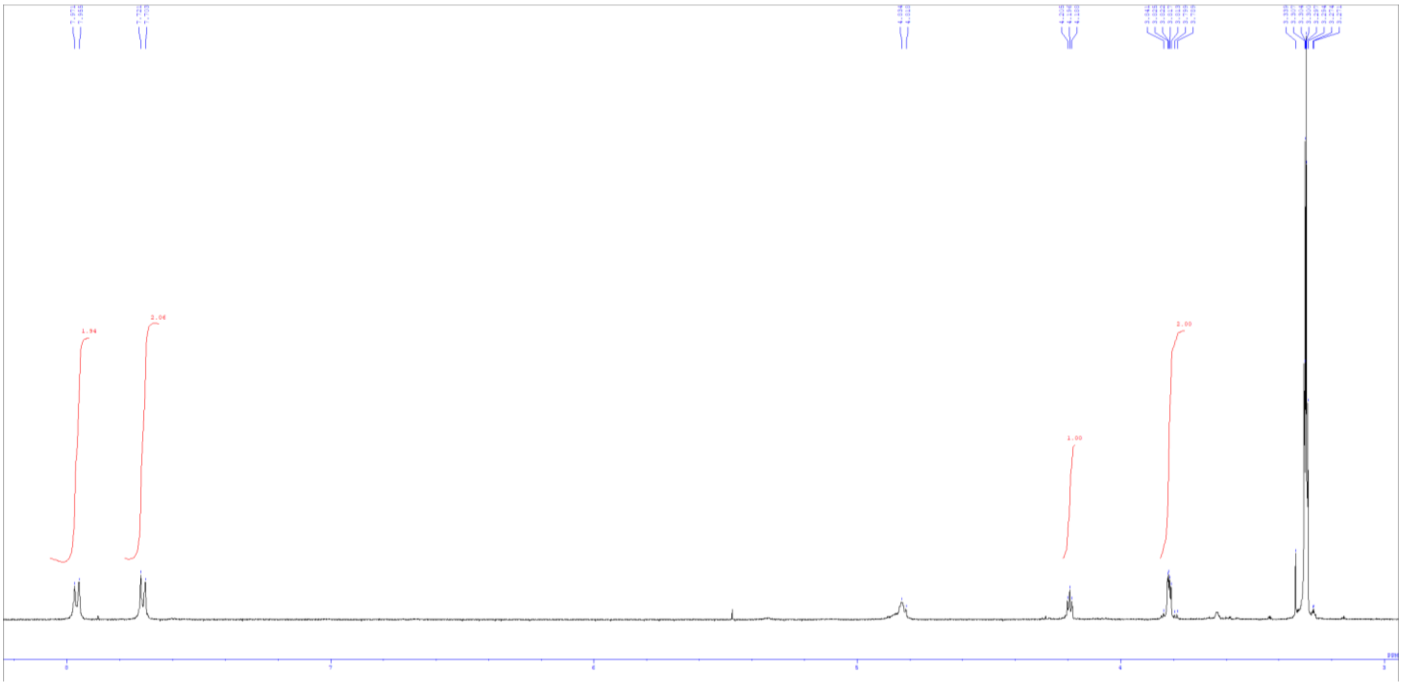

Figure S1. ^{1}H NMR spectrum of **1** (CD_3OD).

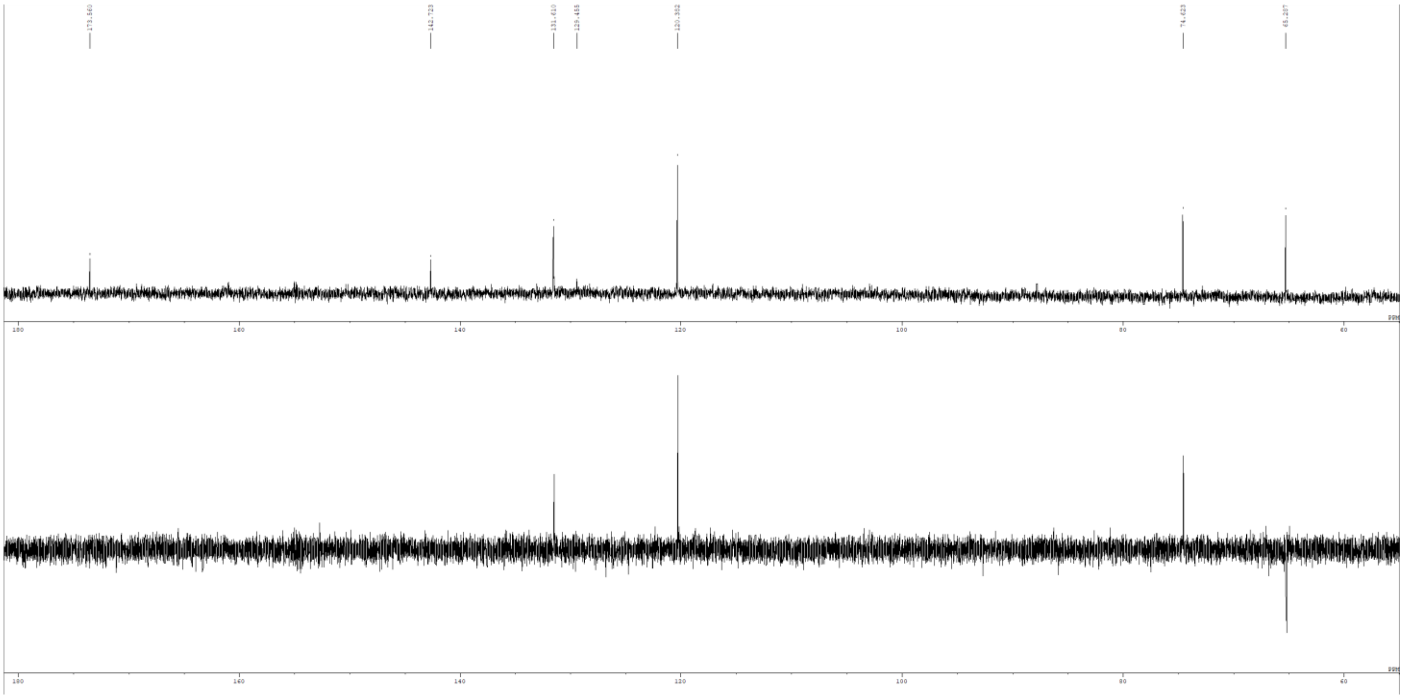

Figure S2. ^{13}C NMR and DEPT spectra of **1** (CD_3OD).

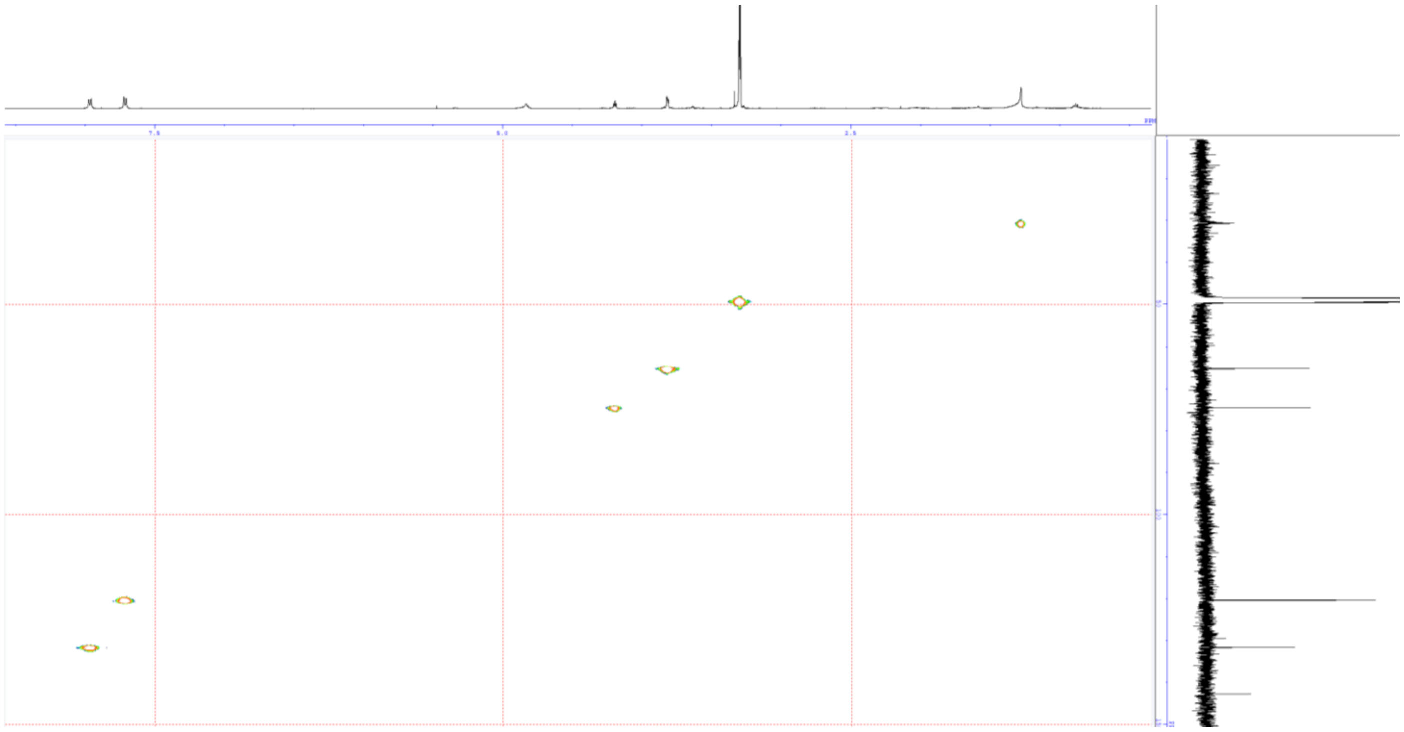

Figure S3. HMQC spectrum of **1** (CD_3OD).

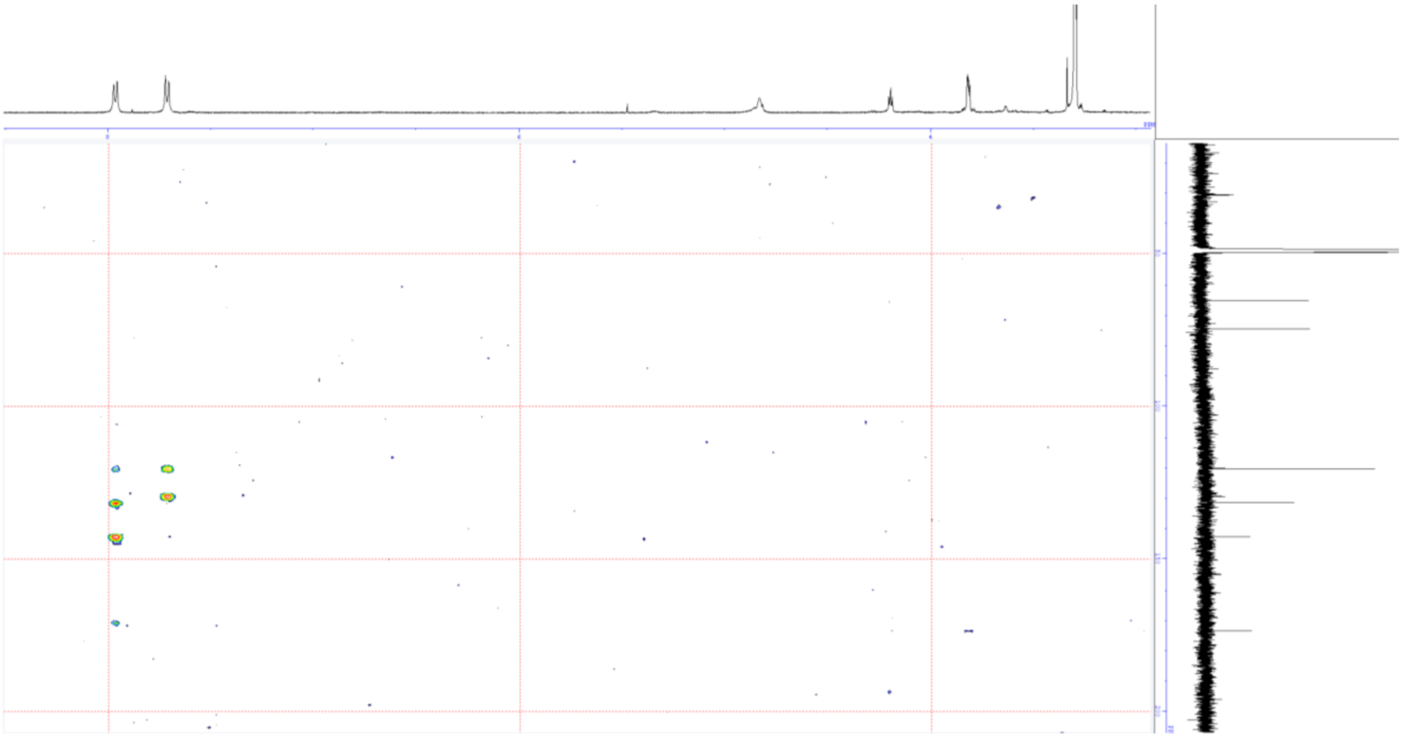

Figure S4. HMBC spectrum of **1** (CD_3OD).

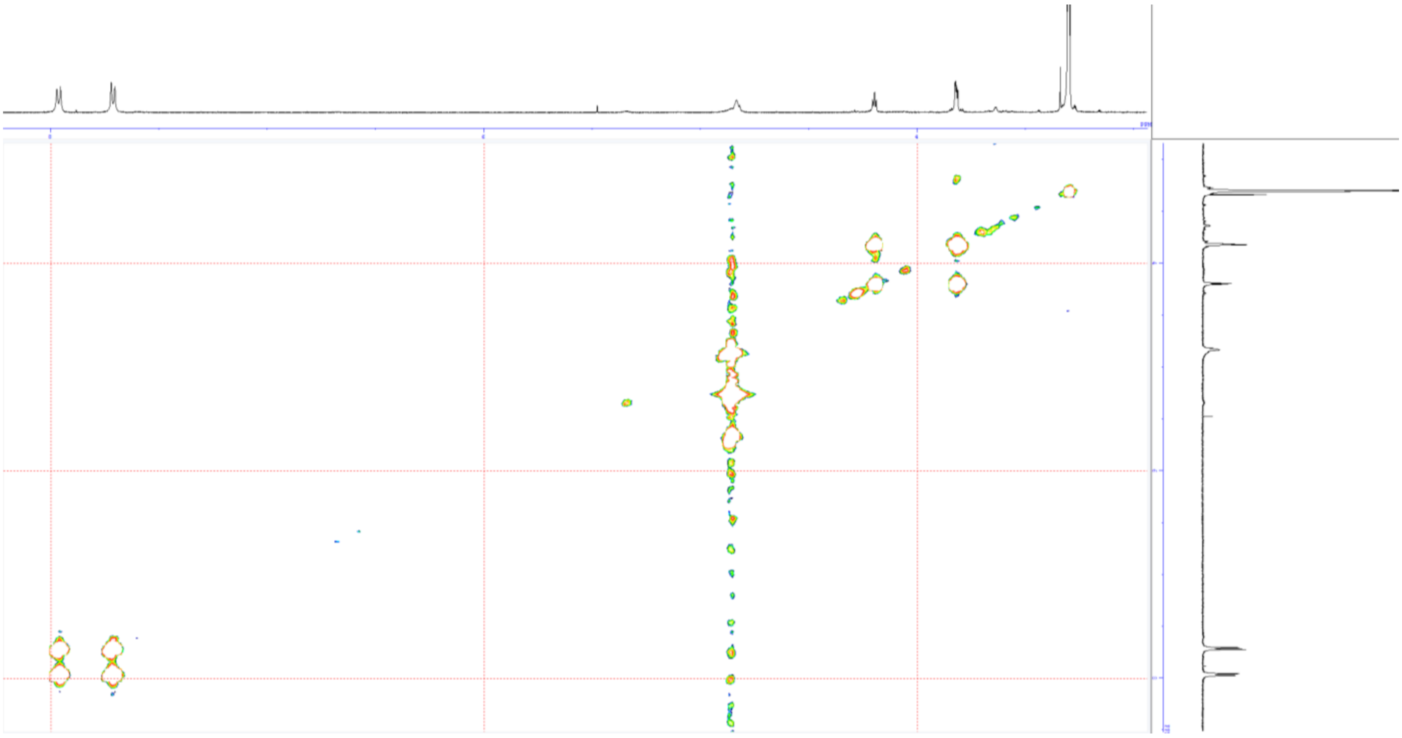

Figure S5. COSY spectrum of **1** (CD_3OD).

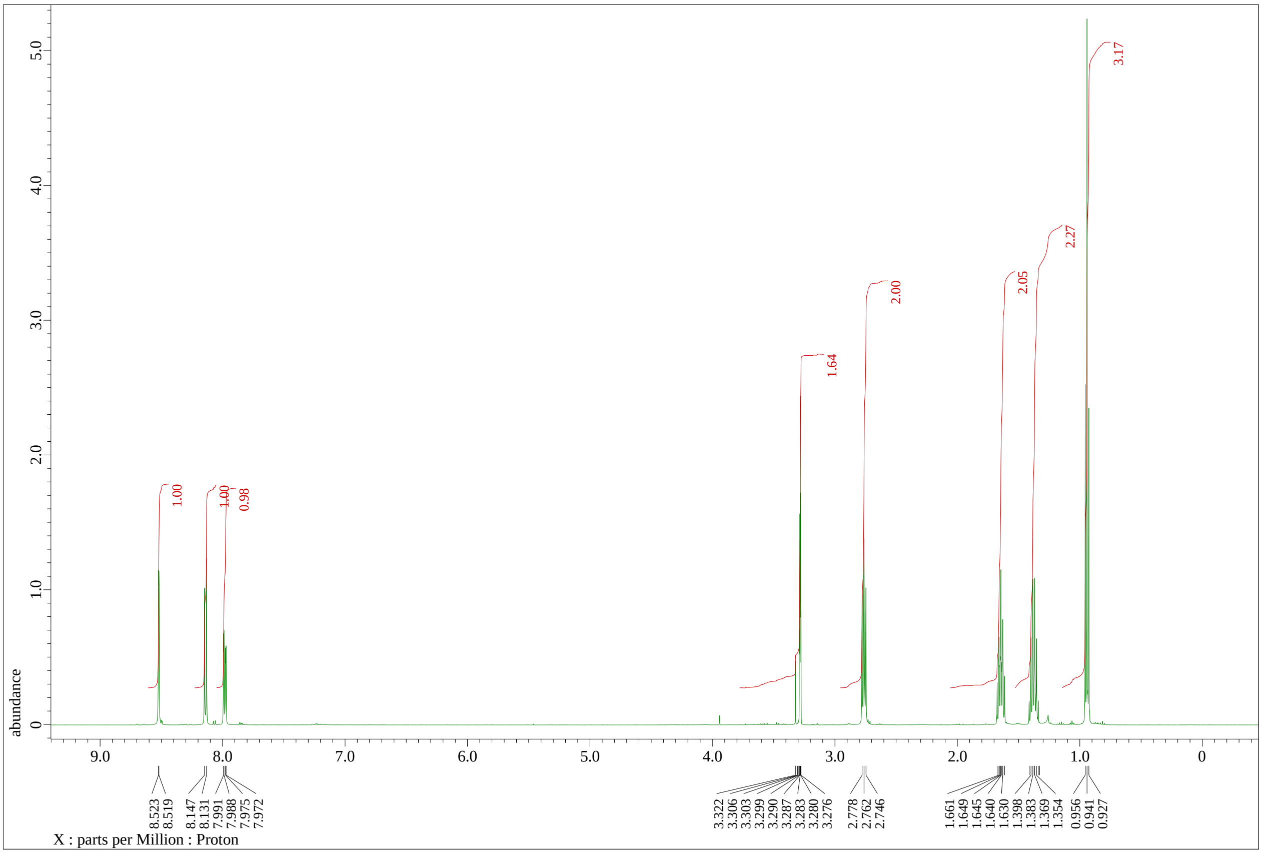

Figure S6. ^{1}H NMR spectrum of **2** (CD_3OD).

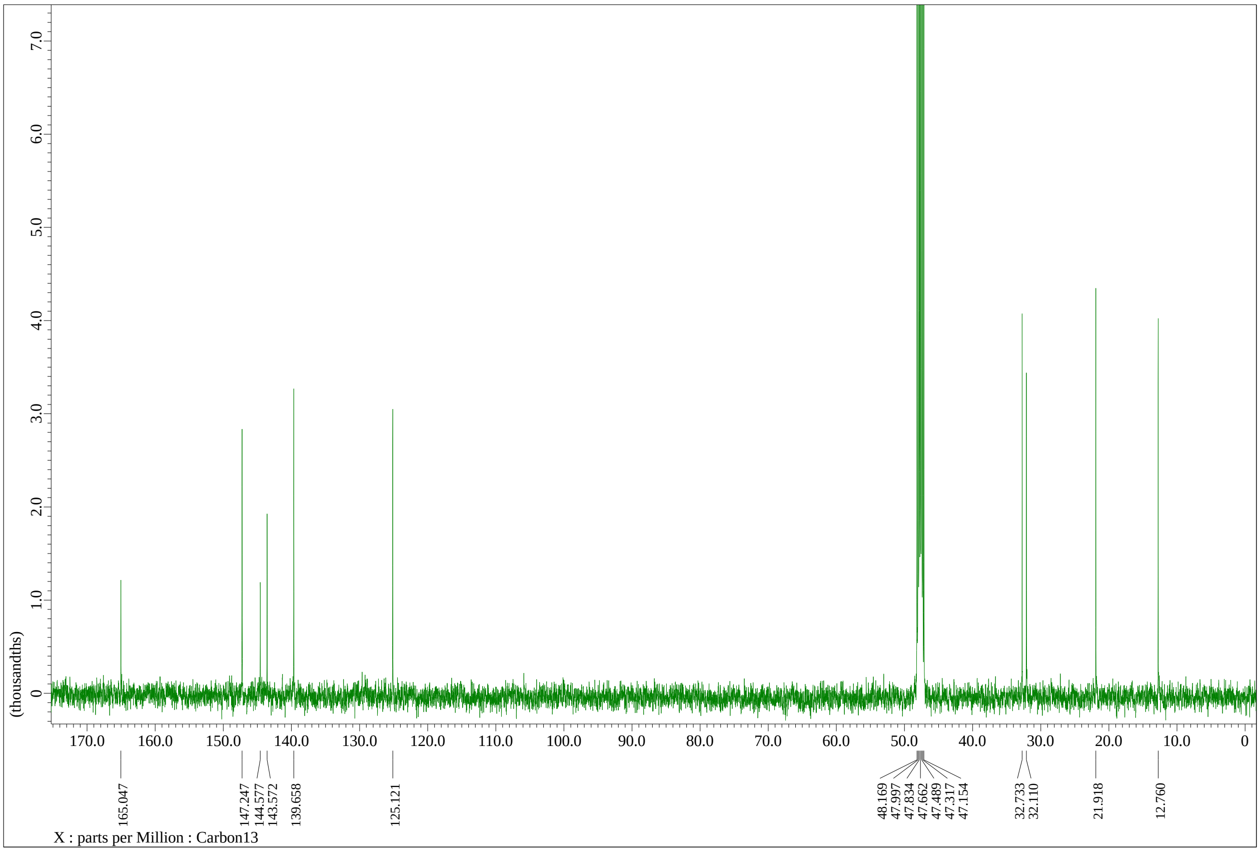

Figure S7. ^{13}C NMR spectrum of **2** (CD_3OD).

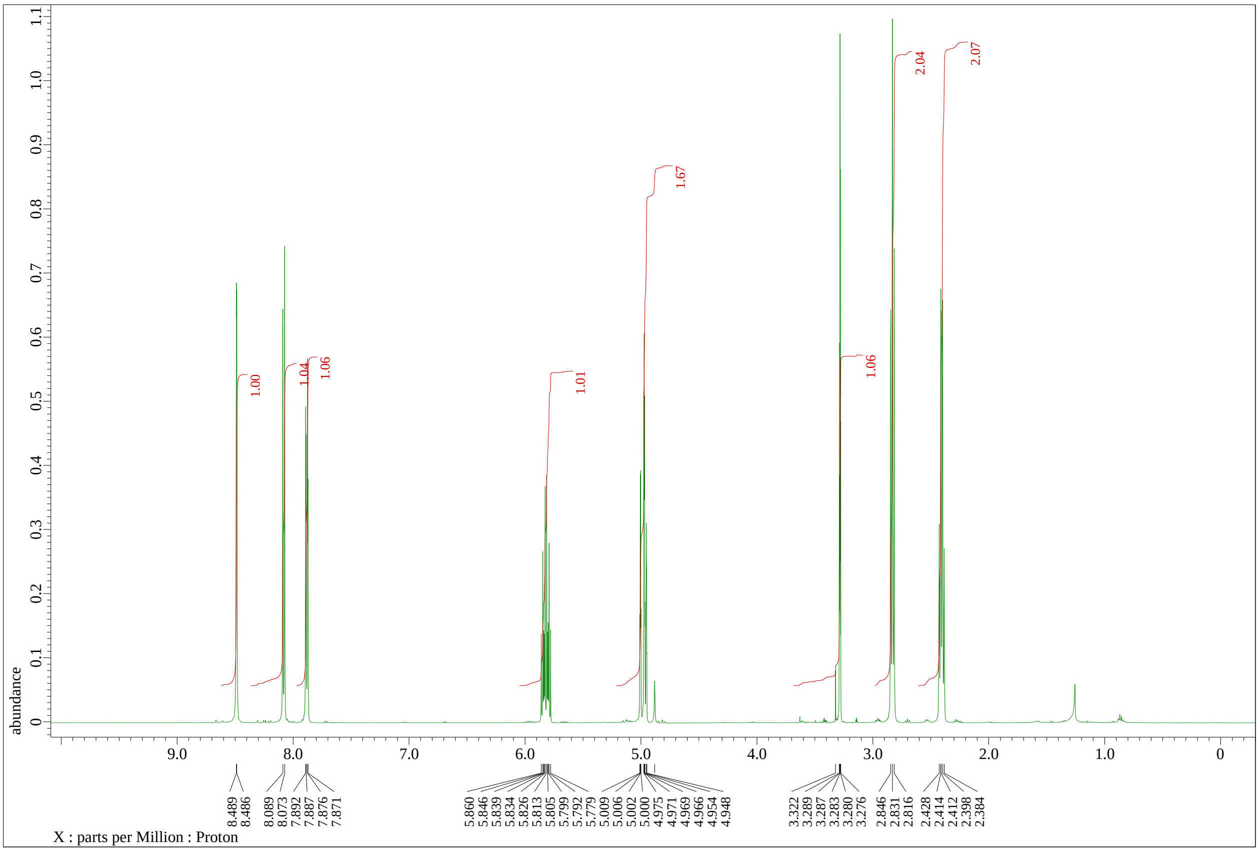

Figure S8. ^{1}H NMR spectrum of **3** (CD_3OD).

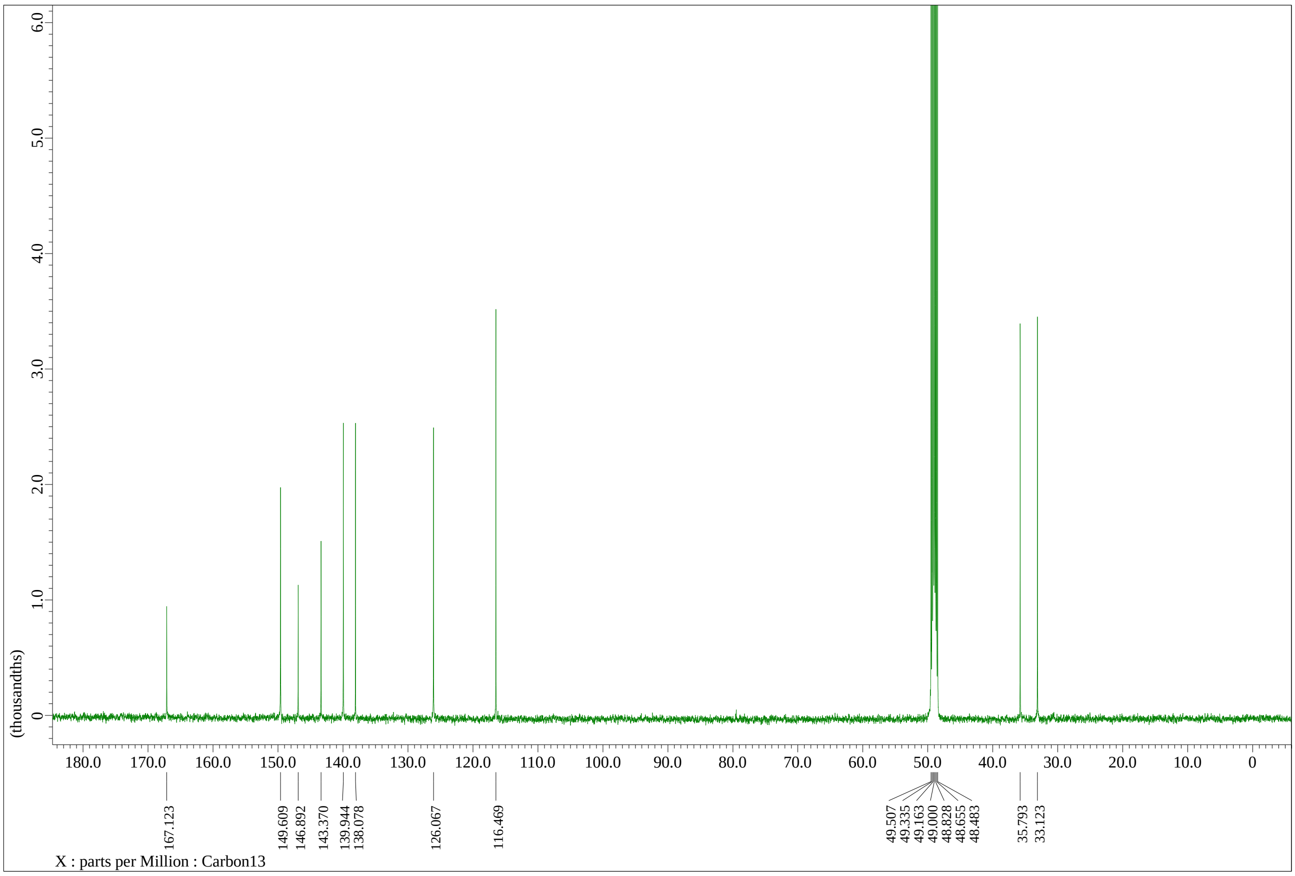

Figure S9. ^{13}C NMR spectrum of **3** (CD_3OD).

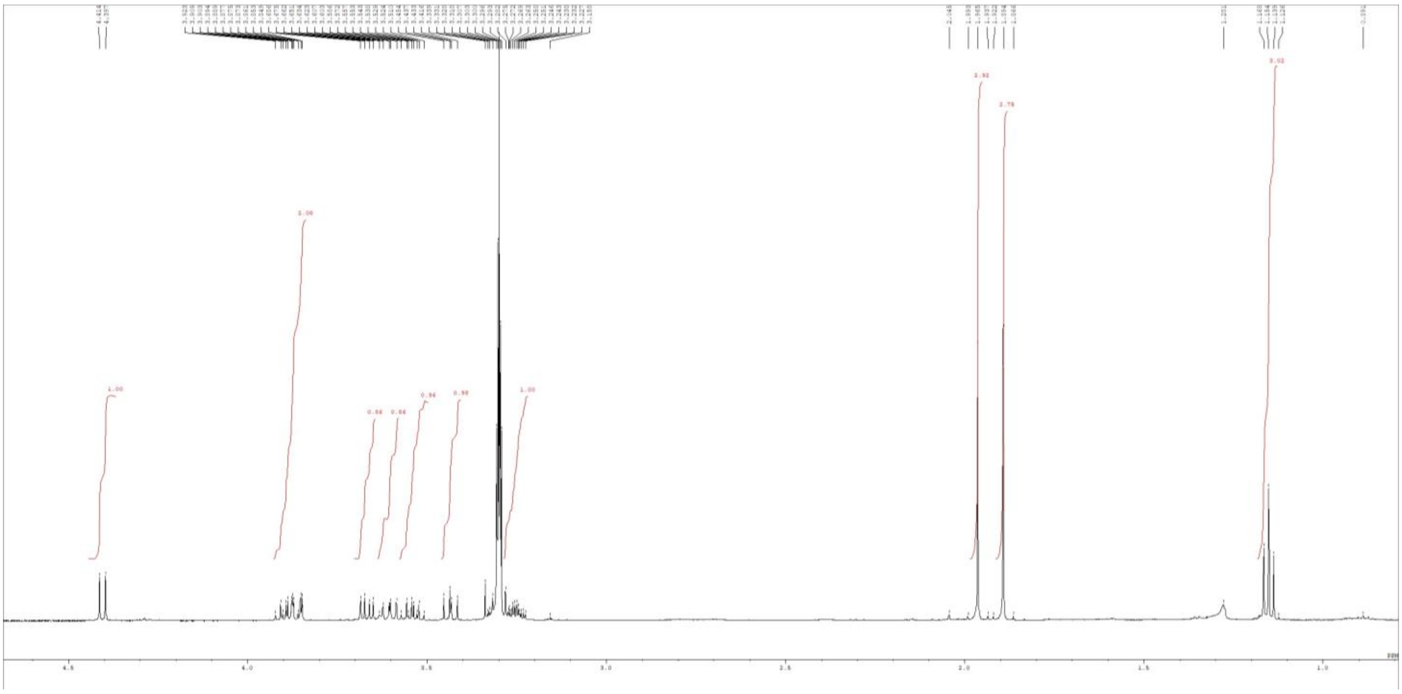

Figure S10. ^{1}H NMR spectrum of **4** (CD_3OD).

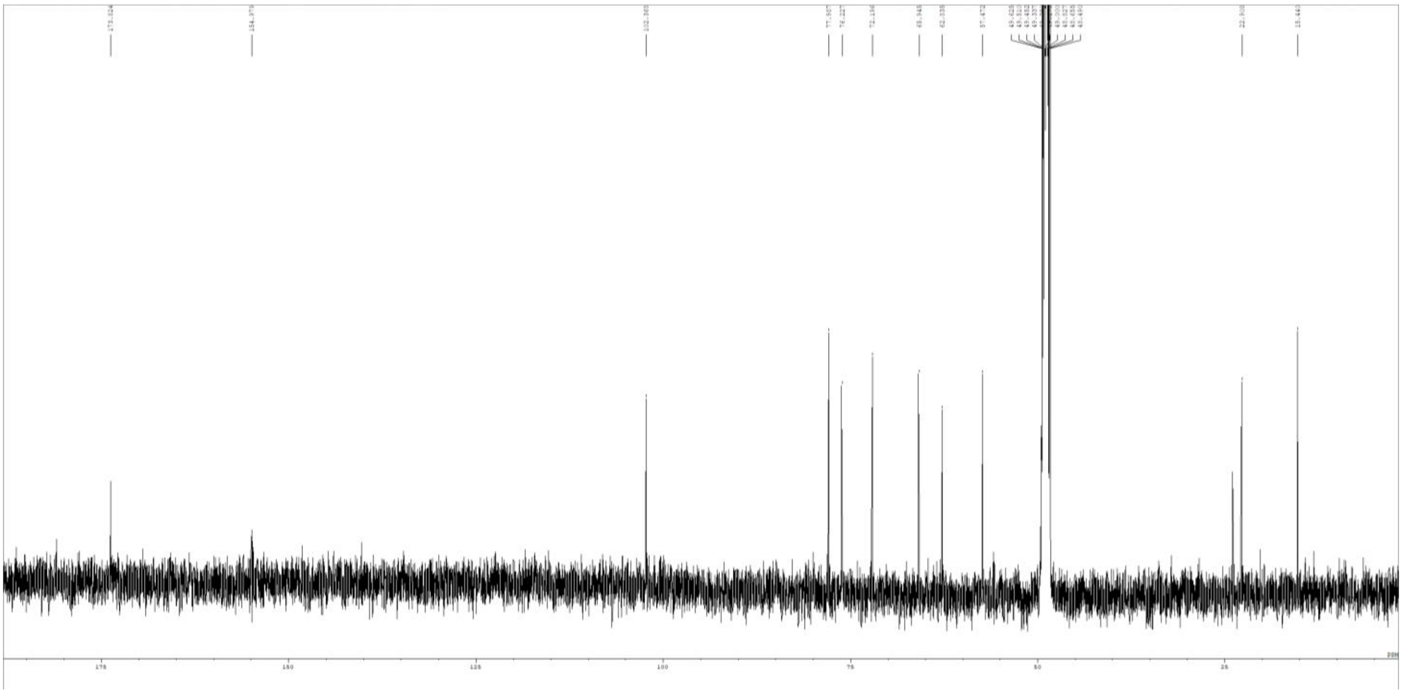

Figure S11. ^{13}C NMR spectrum of **4** (CD_3OD).

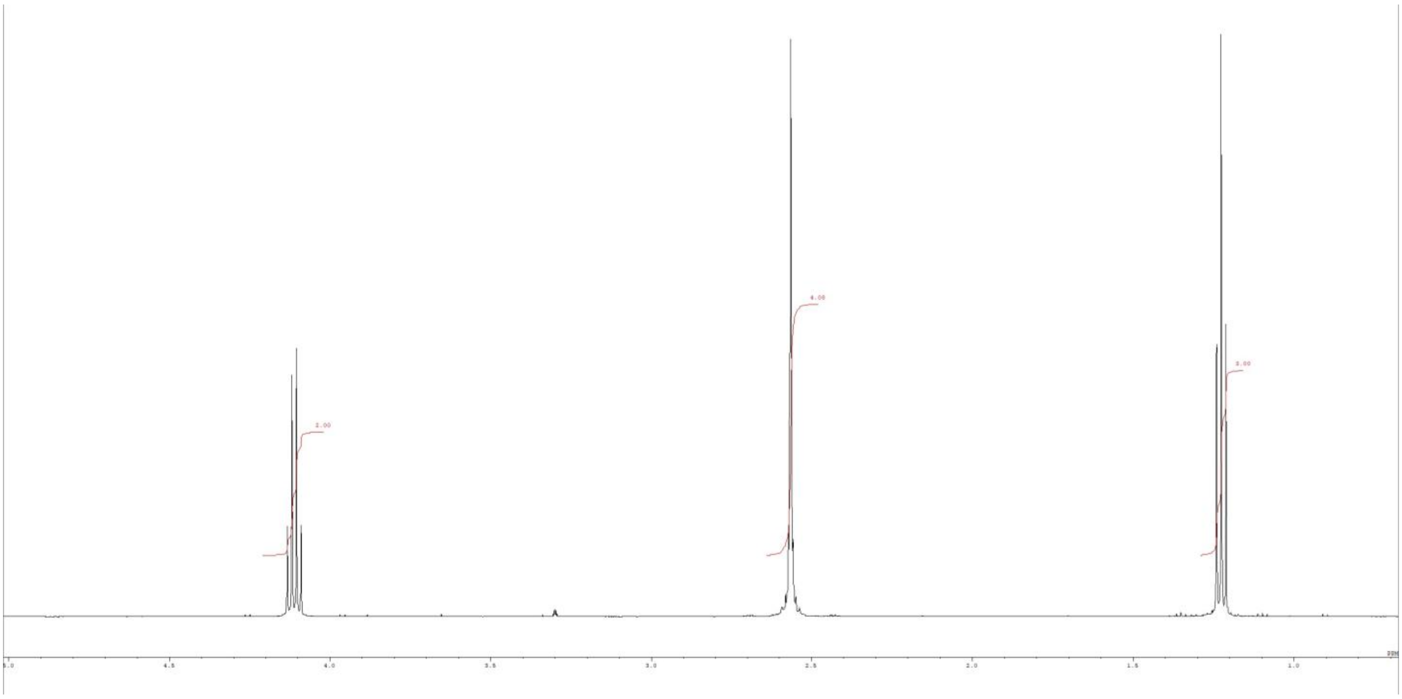

Figure S12. ^{1}H NMR spectrum of **5** (CD_3OD).

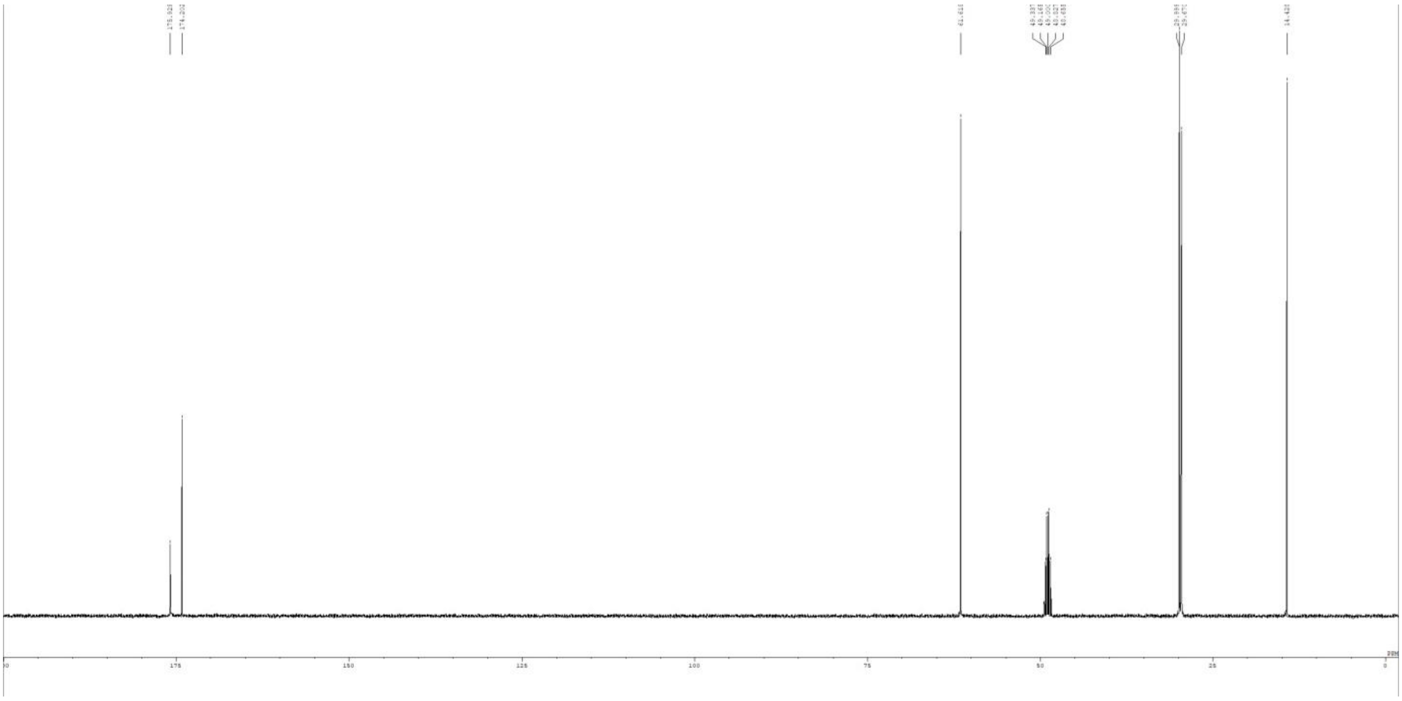

Figure S13. ^{13}C NMR spectrum of **5** (CD_3OD).

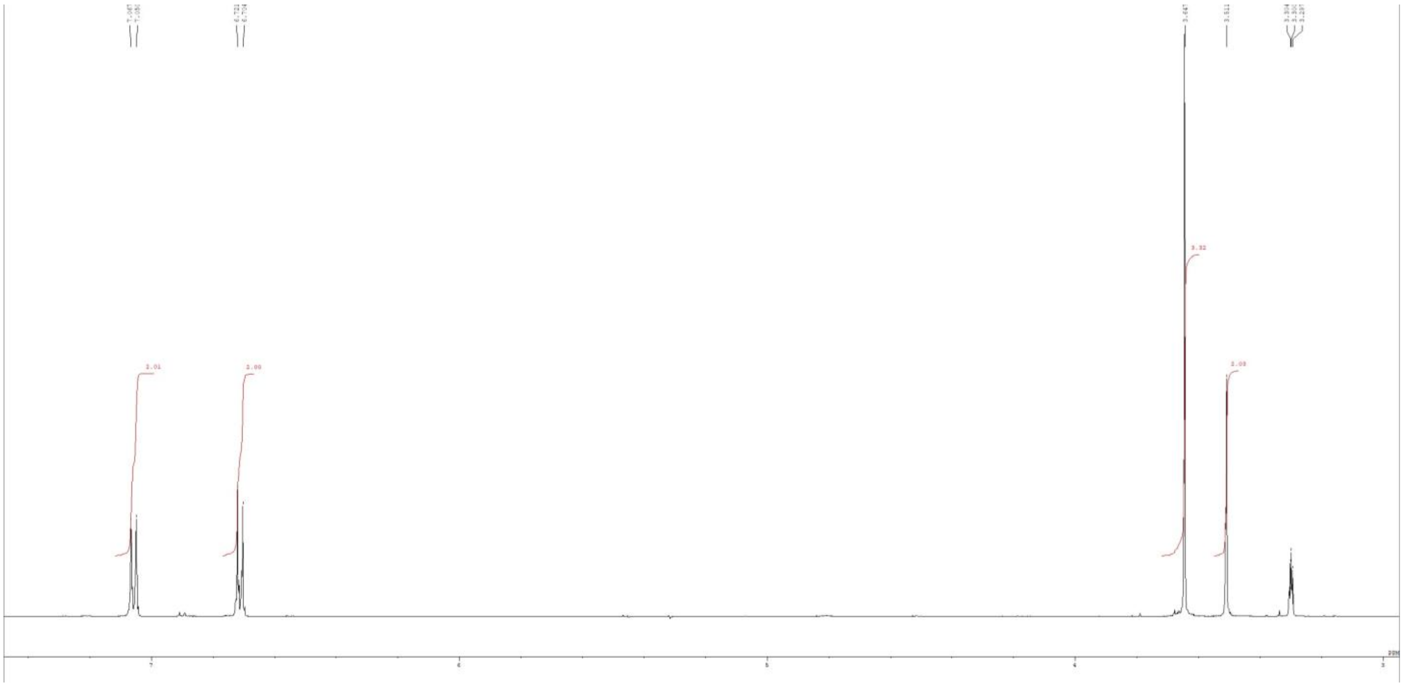

Figure S14. ^{1}H NMR spectrum of **6** (CD_3OD).

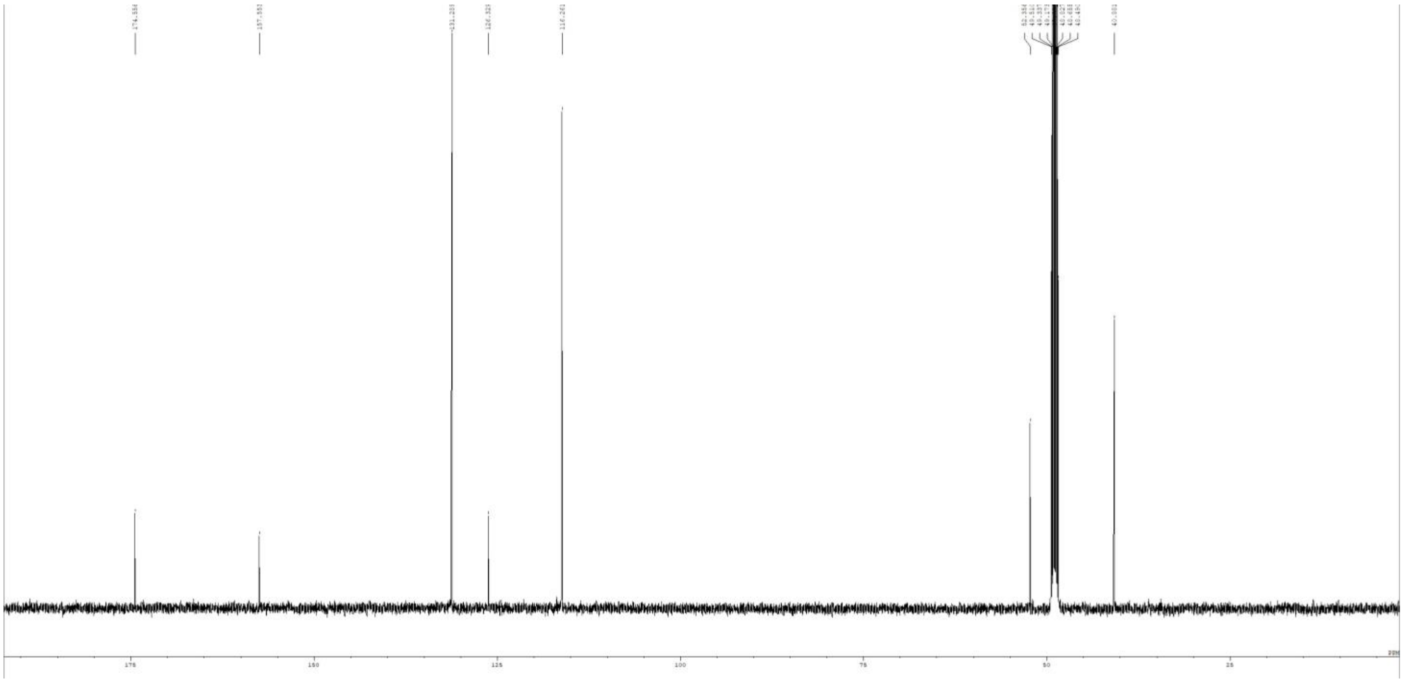

Figure S15. ^{13}C NMR spectrum of **6** (CD_3OD).
